# Multi-Source Remotely Sensed Data Combination: Projection Transformation Gap-Fill Procedure

**DOI:** 10.3390/s8074429

**Published:** 2008-07-29

**Authors:** Ali Darvishi Boloorani, Stefan Erasmi, Martin Kappas

**Affiliations:** 1 Department of Cartography, GIS & RS, Institute of Geography, Goettingen University, Goettingen, Germany. Address: Goldschmidtstr. 5, 37077 Goettingen, Germany; E-mail: adarvis@uni-goettingen.de; Tel: +49(0)551/39-14364; Fax: +49(0)551/39-8020; 2 Ministry of Science Research and Technology of Iran (MSRTI), Bolvare Khordin, Shahrake Gharb, 14665-1513, Tehran, Iran; 3,4 Department of Cartography, GIS & RS, Institute of Geography, Goettingen University, Goettingen, Germany. Address: Goldschmidtstr. 5, 37077 Goettingen, Germany; E-mail: serasmi@uni-goettingen.de^2^, mkappas^3^@uni-goettingen.de; Tel: +49(0)551/39-14364; Fax: +49(0)551/39-8020

**Keywords:** remote sensing, gap-fill, data combination, Principal Component Transformation (PCT)

## Abstract

In this work a new gap-fill technique entitled projection transformation has been developed and used for filling missing parts of remotely sensed imagery. In general techniques for filling missing areas of an image break down into three main categories: first multi-source techniques that take advantages of other data sources (e.g. using cloud free images to fabricate the cloudy areas of other images); the second ones that fabricate the gap areas using non-gapped parts of an image itself, this group of techniques are referred to as single-source gap-fill procedures; and the third group which applies methods that are a combination of both mentioned techniques, therefore they are called hybrid gap- fill procedures. Here a new developed multi-source methodology called “projection transformation for filling a simulated gapped area in Landsat7/ETM+ imagery” is introduced. The auxiliary imagery for filling the gaps is an earlier obtained L7/ETM+ imagery. Quality of the technique was evaluated from three points of view: statistical accuracy measuring, visual comparison, and post classification accuracy assessment. These evaluation indicators are compared to the results obtained from a commonly used technique by the USGS, the Local Linear Histogram Matching (LLHM) [1]. Results show the superiority of our technique over LLHM in almost all aspects of accuracy.

## 1. Introduction

Gapping is a typical phenomenon with remote sensing imagery. (As this occurrence could have dynamic and diverse characteristics thus there are a variety of techniques that could be applied). Construction of gapped areas from satellite imagery is of high interest for visual image interpretation and digital image classification purposes. Imagery gaps can have several reasons, e.g., cloud coverage for optical imagery, shadowed area for SAR data sets, or instrumentation errors e.g. SLC-off problem [1] and line striping [2]; such areas are referred to in this paper as gap areas i.e. the pixels are set to zero (Fig. 2). Gap areas can have different sizes, dimensions, and locations. For instance the striping problem may affect just one column and/or row of pixels while the cloudy area in an image could be more than 50% of a satellite imagery scene [3].

In the literature many methodologies have been proposed for construction of gapped pixels. Generally the applied procedures are categorized into three main groups: (i) multi-source; (ii) single- source; and (iii) hybrid [4]. In the first category gap areas are constructed using other useful sources. For instance [1] used non-gapped images (hereafter referred as fill image) (Fig. 2) for fabricating gapped area. The Local Linear Histogram Matching (LLHM) technique was developed for their procedure. Based on the obtained results if images have specific criteria like minimum temporal shifts, seasonal differences, and land cover changes the filled gaps are reliable and effective. But the problem will be appear when there are sharp differences in target radiances due to land cover changes, atmospheric effects, or sun glint changes [1].

The second category mostly are based on within image pixel similarities/dissimilarity rules (e.g. geostatistical techniques) where gapped areas are constructed using non-gapped areas (Fig. 2) in the image itself. In this category the simplest case is based on the replacement of missing pixel with the values of the neighboring pixels [5]. Geostatistics-based methodologies are other type of the single- source gap-fill procedures, for example [6] using ordinary kriging for reconstructing the gapped area in Landsat/ETM+SLC-off imagery which obtained results that are promising and reliable.

The third category in which the attempt is in synergetic usage of two above mentioned methodologies. Therefore gapped areas are filled using non-gapped areas in the image itself while the interpolation process is restricted to the object borders that came from other data sets. For example [6] adapted a standardized ordinary co-kriging which is particularly useful when samples of the variable to be predicted are sparse. [7] used a multi-scale segment-based approach which utilizes the obtained information from other sources in order to keep the interpolation routine within land surface object bounds; they indicated their technique is useful for applications in regional scales e.g. general land cover and crop-specific mapping, but for the applications that require per-pixel accuracy such as urban characterization or impervious surface mapping, is not a strongly recommended approach.

**Figure 1.**
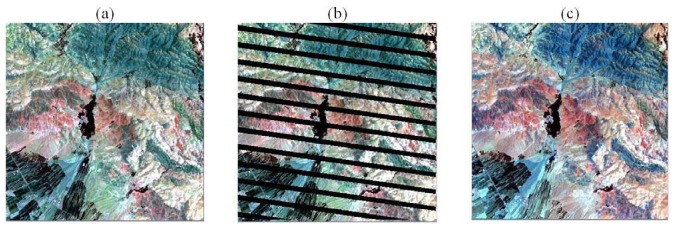
Landsat/ETM+ Satellite images. (a) image from path 166 row 037, acquired may 29, 2000; (b) the simulated SLC-off phenomenon by artificial gap lines in (a); and (c) path 165 row 037, acquired July 07, 1999 (fill image FI).

The objective of this study is to evaluate the capability of a newly developed multispectral projection transformation i.e. PCT gap-fill algorithm in comparison to the commonly used LLHM technique [1]. These two routines are applied and evaluated over a Landsat/ETM+ imagery from Lorestan (Iran) (Fig. 1). The results from the techniques were considered for the statistical and visual properties of the reconstructed area in comparison to the original data. Consequently a statistical indicator as Universal Image Quality Index (UIQI) was used, followed by a post classification accuracy assessment and a visual inspection.

## 2. Satellite Images

Enhanced Thematic Mapper Plus (ETM+) onboard the Landsat 7 remotely sensor satellite was launched on 15 April 1999. It has one panchromatic (520-900 nm), six VNIR/SWIR (450-2350 nm) multispectral, and one thermal (2090-2350 nm) spectral channels. The spatial resolutions of instruments are namely 15m for panchromatic, 30m for six VNIR/SWIR, and 60m for thermal bands. ETM+ as the successor and advanced version of Landsats 4 and 5 was offering valuable data sets to the community of remote sensing and environmental scientists and final users of remote sensing imagery till the malfunctioning of the SLC on May 31, 2003 which interrupted this valuable source of space-borne imagery.

In this work the evaluated data sets are two Landast/7 ETM+ imagery: path 165 row 037, acquired July 07^th^, 1999 (fill image) and path 166 row 037, acquired May 29^th^, 2000 (gap image). The study area is located approximately 20 km south-east of Borujerd, Lorestan (Iran). The image of this area is comprised of 390*390 pixels which are dominated by the typical Zagros sparse oak forests; dry agricultural land; mountain bed rocks and bare soils; a river and sedimentary materials (Fig. 1).

### 2.1. Data preprocessing

For the purpose of topographic distortion reduction [8] the Digital Elevation Model (DEM) with 90 meter spatial resolution (i.e. SRTM topographic data) was used. DEM was resampled to 28.5 meter in **order to** make it compatible to the ETM+ image. With a RMSE less than one pixel, both data sets have been orthogeo-registered. Therefore, expectedly these data will have the same spatial accuracy required for data combination. Rich and valuable knowledge and information are in [9 and 10].

As an elementary step in data combination and gap-fill procedures a precise knowledge of which pixels are valid in an image and which are to be filled is necessary. Therefore data sets must have the highest possible spectral, temporal, radiometric, and spatial consistency. With a view to the fact of temporal changes and differences in the geometry of imaging, absolute radiometric correction as a vital preprocessing step for data fusion and combination processes were carried out. In this regard two steps of data conversion were executed: Quantized DN values to at-sensor radiance transformation [11] and at-sensor radiance to Top-Of-Atmosphere (TOA) reflectance conversion [12]. These two transformations are strongly recommended which will put data from different times and sources into a common radiometric scale. Details of these are provided in [12-14].

## 3. Methodology

### 3.1 Projection transformation

The multidimensionality of remotely sensed imagery provides the possibility of spectral transformations [15]. These spectral projection transformations will make new representative data sets (e.g. components in PCT). The new data set will show an alternative description of the original to which it is related via a mostly linear operation [5]. In the literature exists a branch of spectral projection transformations, like Principal Component Transformation (PCT); Gram-Schmidt; Hue, Saturation, Intensity (HSI); etc. These routines are evaluated for remote sensing image analysis for different applications. For instance reducing data dimensions [16]; improving the visual displaying of imagery; reducing the costs for transmission and storage of data via data compression [17]; and data fusion [18]. For the purpose of this work, PCT as a mathematically lossless and rigorous invertible transformation is selected.

PCT is a mathematically orthogonalizing linear transformation which transforms a multivariate data set to a new coordinate system [2]. A brief description of PCT is outlined and detailed discussion can be found in, for example [16 and 19]. The very simple expression of PCT is

(1) TDS=TM∗ODS

Where TDS is the Transferred Data Set or new components; TM is the Transformation Matrix; and ODS denotes the Original Data Set [16]. The transformation coefficients are found as eigenvectors of the correlation or covariance matrices [2] for the selected bands. In this way the n^th^ principal component is computed as:

(2) $${\text{PC}}_{\text{n}}={\text{SF}}_{\text{n}}\;({\text{m}}_{\text{n}1}{\text{DN}}_{1}+{\text{m}}_{\text{n}2}{\text{DN}}_{2}+\ldots +{\text{m}}_{\text{nn}}{\text{DN}}_{\text{n}}+{\text{M}}_{\text{n}})$$

Where PC_n_ denotes the PC number n; m_nn_ denotes the matrix of transformation coefficients. DN_n_ is the Digital Number of image n; SF_n_ is the adaptable Scale Factor used to distribute the principal components; and M_n_ indicates the adjustable Mean value of principal component n. Over the obtained new components, if inverting transformation be carried out, the original data sets can be recovered with no loss.

### 3.2 PCT-based gap-fill procedure

**Figure 2.**
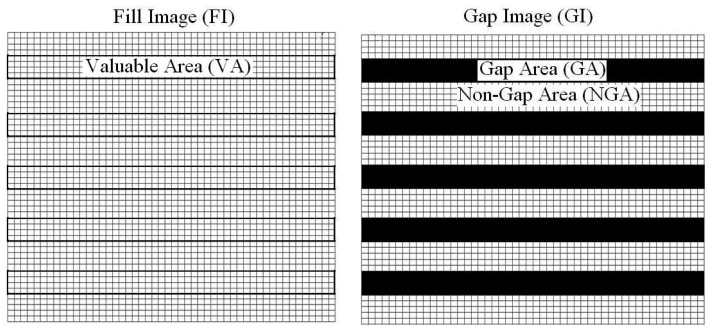
Simulation Landsat/ETM+SLC-off phenomenon.

**Figure 3.**
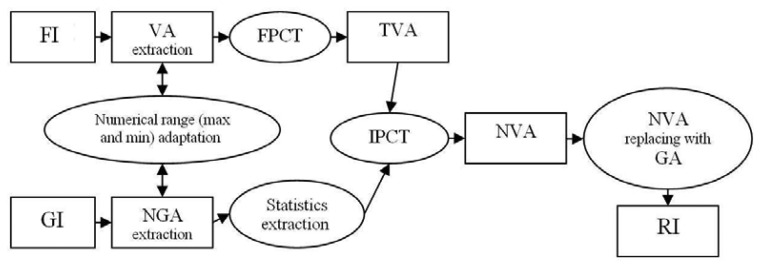
Principal component transformation gap-fill procedure. FI and GI denote the Fill and Gapped Images; VA is the Valuable Area; TVA is the Transformed VA; FPCT and IPCT are the Forward and Inverted Principal Component Transformations; NVA is the New VA; and NGA is the Non-Gap Area (Fig. 2).

The basic idea behind the PCT-gap-fill methodology is to fill gap areas in multispectral images by the help of valuable pixels from the fill images. For the purpose of illustration let FI and VA be the Fill Image and the Valuable Area for fill gaps; GA and NGA be the Gapped Area and Non-Gapped Area from the Gap Image GI (Fig. 2). As shown in Fig. 3 the PCT-gap-fill procedure is based on fore- and backward principal component transformation, the procedure of which will follow the next steps:

(i) pixels in VA numerically adaptation to pixels in NGA;
(ii) using NGAs for calculation the needed statistics for Inverted PCT (IPCT), e.i. the empirical mean, correlation matrix and Eigenvalues;
(iii) VAs conversion to Transformed VAs (TVAs) using the principal component transformation;
(iv) inversely transformation of the TVAs using the obtained statistics from step (ii) to make the New VAs (NVAs); and
(v) replacing the NVAs to the GA to form a Reconstructed Image (RI).

While the first step is always recommended it can be ignored for those cases in which the data sets are radiometrically and atmospherically corrected. For the second step, as the NGA has a very crucial role in the whole process, it must be very carefully selected. Consequently the needed transformation statistics could be obtained from the whole NGA area or some selected samples of it (i.e. the area with minimum differences between two data sets). Generally the selected samples must be the real representative of the physical phenomena from the land surface. The minimum needed pixels for calculation must be provided [5].

For the purpose of VA injection into gapped area, VA pixels must be within the same numerical range (min and max) as NGA pixel values. Therefore this adaptation based on [20] was applied by

(3) $$\text{AVA}=\frac{\left(\text{VA}-\text{MinVA})(\text{MaxNGA}-\text{MinNGA}\right)}{(\text{MinVA}-\text{MinVA})+\text{MinNGA}}$$

Where AVA is Adapted Valuable Area to the Non Gapped Area (NGA) (Fig. 2); VA is Valuable Area; and Min and Max are the minimum and maximum values in both data sets, respectively.

As the obtained statistics from the NGAs are the main parameters of this transformation they must have some necessary characteristics:

(i) they must be the real representative of the physical phenomena, e.g. radimetrically and atmospherically rectified;
(ii) anomaly pixels (e.g. extremely bright or very dark pixels) must be moderated or eliminated; and
(iii) selected NGAs must be similar to the missed pixels as much as possible.

As a consequence, presence of uncommon land covers (e.g. clouds or snows) will shift the calculated statistics (i.e. empirical mean, correlation matrix, and Eigenvalues [5]). In such a situation the calculated NVAs will be more biased to the uncommon land covers classes than the real land cover classes. Accordingly, from the final reconstructed image the NVAs will be brighter or darker than their surrounding pixels.

## 4. Results and Discussion

The selected study area is used to evaluate and compare the results of the PCT and LLHM gap-fill procedures. The used data set is composed of diverse landscapes: the typical Zagros sparse oak forests; dry agricultural land; mountain bed rocks and bare soils; a river and sedimentary materials. The data set from 1999 was used as fill image and the one from 2000 as gap image (which was artificially gapped). Using LLHM and PCT the gapped areas were filled and results were evaluated. From the quality assessment point of view and based on the theory of data fusion the fabricated images can be evaluated visually, spectrally, and spatially. As the GI and FI both have the same spatial resolution (i.e. 28.5 m) spatial comparison is negligible. Accordingly the next three kinds of comparisons were carried out: post classification accuracy assessments; statistical image quality evaluation; and visual investigation [21].

These evaluations are conducted by comparing the fabricated pixels with the original pixel values artificially removed in the SLC-off phenomenon simulation.

**Figure 4.**
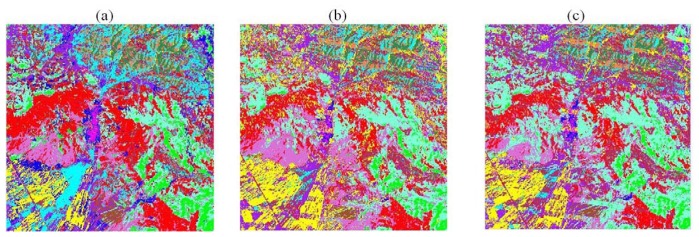
Maximum likelihood classification. (a) original (2000 data set); (b and c) reconstructed data set by PCT and HM method, respectively.

One of the main purposes of the gap fill process is filling the gapped satellite imagery to classify land surface classes. Therefore the fabricated data were evaluated from a post classification accuracy point of view. Maximum likelihood classification as the most common supervised classification method [2] was carried out to classify the reconstructed image. Using the confusion matrix [15], kappa coefficient and overall classification accuracy (as the measures of techniques performance [5]) were adapted (Fig. 4 and Table 1). As can be seen the two classified results are almost the same for the almost unchanged land covers (central parts). But the superiority of PCT is obvious in the areas with high changes in land covers, for example in the lower left parts as agricultural fields and upper right parts as forest area. The fact that the filled lines are seeable in both (Fig. 4(b and c)) but the striping problem has slightly more been alleviated in PCT gap-fill procedure. Also the statistical measurements of the post classification show the superiority of the PCT over LLHM (Table 1).

Using Universal Image Quality Index (UIQI) [22] the performance of the techniques with respect to the spectral information contents of the fabricated pixels in comparison to the original pixel values were measured. This indicator provides a measure of the closeness between fabricated and original areas by utilizing the differences in the statistical distributions of DNs.

(4) $$\text{UIQI}(\text{O},\text{F})=\frac{\delta_{\text{FO}}}{\delta_{\text{F}}\delta_{\text{O}}}\frac{2\mu_{\text{F}}\mu_{\text{O}}}{{\left(\mu_{\text{F}}\right)}^{2}+{\left(\mu_{\text{O}}\right)}^{2}}\frac{2\delta_{\text{F}}\delta_{\text{O}}}{\delta_{\text{F}}^{2}\delta_{\text{O}}^{2}}$$

Where, UIQI is the measure of similarity between the Fabricated (F) and the Original (O) pixels in the gapped area. ^δ_FO_^, ${}^{\delta_{\text{F}}^{2}}$ and ${}^{\delta_{\text{O}}^{2}}$ are the covariance and variances of F and O, respectively. ^μ_F_^ and ^μ_O_^ are the means of F and O, respectively. This index was developed by modeling all possible distortions of an image. It is a combination of three important factors of image impairments including loss of correlation, luminance distortion, and contrast distortion [22]. As most applied gap-fill techniques are performing well in the un-changed areas the high performance of a technique will be apparent in the areas with the highest degrees of changes. Thus using this indicator all possible distortions are evaluated: overall UIQI; UIQI in almost unchanged land surface classes (e.g. soil); and UIQI in the most changed land surface classes (e.g. agricultural fields).

For the purpose of the first evaluation the whole fabricated areas (i.e. 44516 pixels) and for the second one only soil and agricultural land surface classes with 644 pixels and 1042 pixels, respectively, are evaluated.

As can be seen (Table 2), except for bands number 5 and 7 where the differences are slight, in all other bands the results from PCT is remarkably better than LLHM.

**Figure 5.**
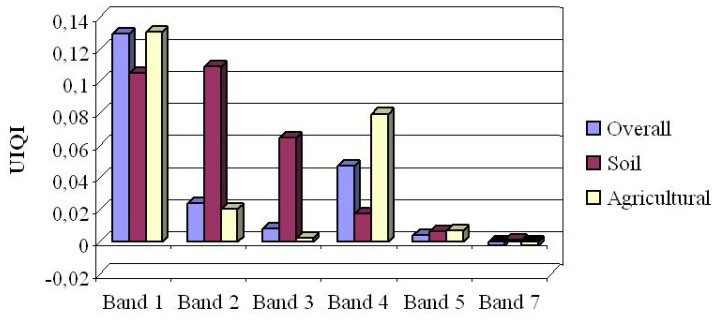
Difference (divergence) between UIQIs for PCT and LLHM. Note: the values are based on the difference: PCT-LLHM, therefore the positive and higher values mean the superiority of PCT over LLHM.

For illustrative purposes the differences (or divergence) between evaluations are calculated through subtraction the UIQI values of LLHM from those of PCT (Table 2(d)). Consequently the positive higher values indicate the superiority of the PCT over LLHM while the negative values indicate the better performance of LLHM. Also the graphical representation of the differences in figure 5 shows that in almost all cases PCT has done better than LLHM. Remarkably, PCT has done better in the visible bands (i.e. 1, 2, 3, 4 bands), but in the shortwave infrared bands (5 and 7) in general both techniques have gotten almost the same accuracies (Table 2(d) and Fig. 5).

**Figure 6.**
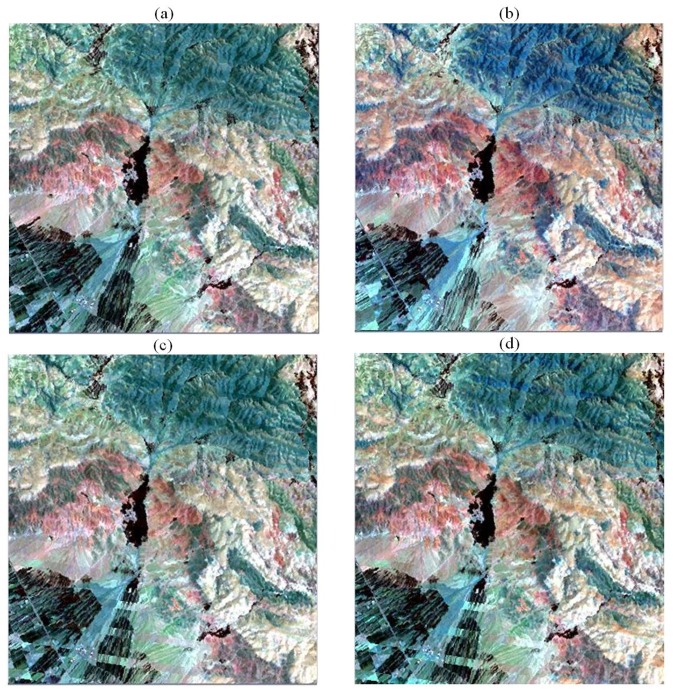
RGB color composite (bands: 7, 3, 2) of the original and fabricated images. (a) original data set (2000); (b) auxiliary (i.e. fill image) data set (1999); (c) fabricated by PCT; and (d) the results of LLHM gap-fill technique.

In the visual inspection (Fig. 6), in comparison to the original image, the obtained results mostly support the findings from two above mentioned evaluations. In the fabricated data sets the areas with lowest spectral changes (e.g. soil and bedrocks) appear approximately the same for both techniques. Therefore, the fabricated gap lines generally look like the original data set. But for the area with sharp differences in the spectral properties of surface materials e.g. agricultural and forest areas the superiority of PCT over LLHM is noticeable.

In figure 6 the fill image (b) color in the upper right parts (forest area) is blue while in the gapped image (a) these areas are green. From the visual comparison points of view the reconstructed lines by the PCT (c) are greener and more similar to their surrounding pixels, while those fabricated by LLHM are more similar to the fill image therefore the inconsistency is more visible. Also in another parts of the images constructed by LLHM the influence of the fill image is more visible than in those constructed by PCT.

## 5. Conclusion

In this paper PCT-based gap-fill approach for filling the gaps in a simulated Landsat/ETM+SLC-off image was presented. This approach uses a fore- and backward principal component transformation over auxiliary imagery to fill the gapped area while the needed statistics for these transformations came from non-gap areas. Obtained results determine that the PCT-gap-fill method, in general, can preserve the radiometric characteristics of multispectral data sets to a higher extent than the LLHM- gap-fill procedure. Further superiority of this techniques is in the areas with sharp radiometric changes e.g. in areas with vegetation land cover, for which the spectral preservation is higher than that of LLHM. The results from the classified data indicate that this technique will be useful for land surface classification and land cover mapping (e.g. forest, soil) although the gap lines are still visible in areas with sharp radiometric differences. Therefore, for applications that require per-pixel accuracy and also for areas with highly dynamic properties e.g. agricultural fields and urban areas, this technique must be used carefully. Finally it must kept in mind that this procedure is sensitive to very high and low pixel values (e.g. cloud covers and water bodies) in the gapped image. Those values will affect the whole process and consequently cause bias of the reconstructed area to very high or low radiometric values. However, if the selected pixels (i.e. NGAs for statistical parameters calculation) are radiometrically and atmospherically corrected and the sharply different pixels are removed, the PCT will provide a very good accuracy and performance.

## Figures and Tables

**Table 1.** Post classification accuracy assessment.

Technique	Overall accuracy (percent)	Kappa coefficient
PCT	73.6729	0.7021
LLHM	61.1466	0.5618

**Table 2.** Universal Image Quality Index obtained from the comparison of the original and fabricated multispectral images. (a) the UIQI for PCT and LLHM techniques through all reconstructed areas; (b) the UIQI for LLHM for soil and agricultural areas as the lowest and highest changed land cover classes, respectively; (c) the same as (b) for the PCT gap-fill procedure; and (d) the differences between PCT and LLHM in the three earlier evaluations, given as D=PCT-LLHM. The positive and higher values show the superiority of PCT over LLHM. B columns indicate the band numbers. Note: numbers are 3 decimal rounded.

(a)			(b)			(c)			(d)			
B	PCT	LLHM	B	Soil	Agr.	B	Soil	Agr.	B	Overall	Soil	Agr.
			
1	**0.85**	**0.72**	1	**0.76**	**0.65**	1	**0.86**	**0.78**	1	**0.13**	**0.10**	**0.13**
2	**0.86**	**0.84**	2	**0.79**	**0.76**	2	**0.90**	**0.78**	2	**0.02**	**0.10**	**0.02**
3	**0.86**	**0.86**	3	**0.83**	**0.77**	3	**0.89**	**0.77**	3	**0.00**	**0.06**	**0.00**
4	**0.87**	**0.82**	4	**0.89**	**0.67**	4	**0.91**	**0.75**	4	**0.05**	**0.02**	**0.08**
5	**0.88**	**0.87**	5	**0.89**	**0.72**	5	**0.90**	**0.73**	5	**0.01**	**0.01**	**0.01**
7	**0.87**	**0.87**	7	**0.87**	**0.74**	7	**0.87**	**0.74**	7	**-0.00**	**0.00**	**-0.00**
